# Machine learning-based CT radiomics model distinguishes COVID-19 from non-COVID-19 pneumonia

**DOI:** 10.1186/s12879-021-06614-6

**Published:** 2021-09-08

**Authors:** Hui Juan Chen, Li Mao, Yang Chen, Li Yuan, Fei Wang, Xiuli Li, Qinlei Cai, Jie Qiu, Feng Chen

**Affiliations:** 1 Department of Radiology, Hainan General Hospital (Hainan Affiliated Hospital of Hainan Medical University), No. 19, Xiuhua St, Xiuying Dic, Haikou, Hainan 570311 People’s Republic of China; 2 Deepwise AI Lab, Deepwise Inc., No. 8 Haidian avenue, Sinosteel International Plaza, Beijing, 100080 China; 3 Department of Ultrasound, Hainan General Hospital (Hainan Affiliated Hospital of Hainan Medical University), No. 19, Xiuhua St, Xiuying Dic, Haikou, Hainan 570311 People’s Republic of China

**Keywords:** Machine learning, Radiomics, Coronavirus Disease 2019 (COVID-19), Non-COVID-19 pneumonia

## Abstract

**Background:**

To develop a machine learning-based CT radiomics model is critical for the accurate diagnosis of the rapid spreading coronavirus disease 2019 (COVID-19).

**Methods:**

In this retrospective study, a total of 326 chest CT exams from 134 patients (63 confirmed COVID-19 patients and 71 non-COVID-19 patients) were collected from January 20 to February 8, 2020. A semi-automatic segmentation procedure was used to delineate the volume of interest (VOI), and radiomic features were extracted. The Support Vector Machine (SVM) model was built on the combination of 4 groups of features, including radiomic features, traditional radiological features, quantifying features, and clinical features. By repeating cross-validation procedure, the performance on the time-independent testing cohort was evaluated by the area under the receiver operating characteristic curve (AUC), accuracy, sensitivity, and specificity.

**Results:**

For the SVM model built on the combination of 4 groups of features (integrated model), the per-exam AUC was 0.925 (95% CI 0.856 to 0.994) for differentiating COVID-19 on the testing cohort, and the sensitivity and specificity were 0.816 (95% CI 0.651 to 0.917) and 0.923 (95% CI 0.621 to 0.996), respectively. As for the SVM models built on radiomic features, radiological features, quantifying features, and clinical features, individually, the AUC on the testing cohort reached 0.765, 0.818, 0.607, and 0.739, respectively, significantly lower than the integrated model, except for the radiomic model.

**Conclusion:**

The machine learning-based CT radiomics models may accurately classify COVID-19, helping clinicians and radiologists to identify COVID-19 positive cases.

**Supplementary Information:**

The online version contains supplementary material available at 10.1186/s12879-021-06614-6.

## Background

Coronavirus disease 2019 (COVID-19) has spread throughout the world widely and rapidly since late December 2019 [1, 2]. The newly emerging disease is highly contagious and may cause severe acute respiratory distress or multiple organ failure in severe cases [3–6]. The World Health Organization (WHO) declared the outbreak of COVID-19 as a “public health emergency of international concern” (PHEIC) on January 30, 2020.

At present, the gold standard for the diagnosis of COVID-19 is reverse-transcription polymerase chain reaction (RT-PCR). However, the high false-negative rate [7] and the shortage of RT-PCR assay in the early stage of the outbreak limited the early detection and treatment of the presumptive patients [8, 9]. This speeded up the spread of COVID-19. Therefore, fast diagnosis is important for controlling the spread of COVID-19. Recent studies have demonstrated that computed tomography (CT), as a non-invasive imaging approach, is of great value in detecting lung lesions in patients with COVID-19 infection [2, 10]. Besides, CT had much higher sensitivity than initial RT-PCR in diagnosing COVID-19 [8, 9]. Consequently, CT could be used as an effective tool for early detection and diagnosis of COVID-19. We should not neglect the fact that COVID-19 may have certain similar CT imaging features with other types of pneumonia, thus making it hard to differentiate. Although measures are taken to control the spread of the disease, there have been 176,531,710 confirmed cases of COVID-19 globally, including 3,826,181 deaths, till 11:32 am CEST, 17 June 2021. Concerning the pandemic, accurate and fast diagnosis of COVID-19 is vital to isolate infected patients and slow down the spread of this disease.

Current studies have demonstrated that artificial intelligence could distinguish COVID-19 from other pneumonia [11, 12], improving radiologists’ performance in distinguishing COVID-19 from non-COVID-19 pneumonia on chest CT and providing clinical prognosis with good accuracy that can assist clinicians to adjust their clinical management timely and allocate resources appropriately [13–19]. However, CT manifestations of COVID-19 resemble other types of viral pneumonia such as severe acute respiratory syndrome coronavirus and Middle East respiratory syndrome coronavirus. Additionally, the non-COVID-19 diseases included as a comparison group are long before the COVID-19 outbreak [20]. Since the CT manifestations of common pneumonia resemble those of COVID-19 pneumonia, the most difficult situation in clinical diagnosis and treatment is to identify other types of pneumonia that occur in the same period as the outbreak of COVID-19.

In recent years, much attention has been paid to radiomics in diagnosing diseases and evaluating treatment outcomes [21, 22]. Specifically, radiomics is of great value in medical imaging because of its ability to extract high throughput quantitative descriptors from routine computed tomography (CT) studies [22]. Radiomics has been applied to many areas of cancer research, such as tumor detection, preoperative prediction of lymph node metastasis, and therapeutic response assessment [21, 23, 24]. Recently, radiomics has been proved to be helpful in COVID-19 screening, diagnosis, prediction the length of hospital stay, and assessment of the imaging characteristics and risk factors associated with adverse composite endpoints in patients with COVID-19 pneumonia [25–28]. Radiomics is also useful in the identification of COVID-19 [29, 30], differentiating clinical types of COVID‑19 [31], and the prediction of poor prognostic outcomes in COVID-19 [32]. Recently, CT radiomics was found to perform better in the accurate diagnosis of COVID-19 pneumonia compared with the COVID-19 reporting and data system [33]. However, these studies were limited in a small sample size. In the study of Qi et al., a total of 31 patients were included in the study [26]. Some did not extract high-throughput imaging features [28]. Besides, few studies have been done including holistic analysis of different radiomics features regarding COVID-19. The purpose of this study was to develop and test machine learning-based CT radiomics models including different radiomics features for the classification of COVID-19.

## Methods

### Study population

**Fig. 1:**
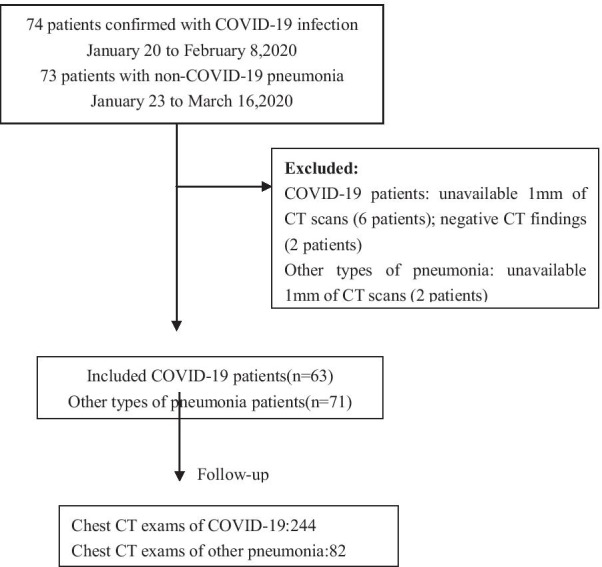
Flowchart of this study

This retrospective study was waived by the ethics committees of the Hainan General hospital. In total, 74 patients confirmed with COVID-19 infection from January 20 to February 8, 2020, and 82 patients with other types of pneumonia in the corresponding period were collected. In the COVID-19 dataset, 63 patients who met the following inclusion criteria were finally included: (i) RT-PCR confirmed COVID-19; (ii) non-contrast CT at diagnosis time; (iii) positive CT findings. 71 patients with non-COVID-19 pneumonia who met the following inclusion criteria were included: (i) RT-PCR excluded COVID-19; (ii) non-contrast CT at diagnosis time; (iii) pneumonia highly suspected with COVID-19 by CT. The exclusion criteria were as follows: (1) contrast CT exams; (2) exams without slice thickness of 1 mm; (3) negative CT findings. Finally, 326 chest CT exams from 134 patients were included in this study (Fig. 1). The average age was 47.0 ± 15.4 years. Specifically, we included 244 (75%) exams for COVID-19 and 82 (25%) for non-COVID-19 pneumonia in the study.

All the patients with COVID-19 were confirmed as positive by RT PCR and were acquired from January 21, 2020, to Feb 8, 2020. The most common symptoms were fever (82%) and cough (77%). Each patient had one or multiple CT scans during the progression of the disease. The follow-up study was continued until February 19, 2020.

Other types of pneumonia patients over the corresponding period between January 23 to March 16, 2020 were selected from the same hospital. For 82 patients with negative RT-PCR results, pneumonia was diagnosed according to the Infectious Diseases Society of America/American Thoracic Society (IDSA/ATS) guidelines [34]. Patients with at least one of the following clinical symptoms: cough, sputum, fever, dyspnea, and pleuritic chest pain, plus at least one finding of coarse crackles on auscultation or elevated inflammatory biomarkers, in addition to a new pulmonary infiltration on chest CT, would be diagnosed to be infected with pneumonia. The admission distribution of the patients with other types of pneumonia was: outpatient (86%, 61 of 71), inpatient (14%, 10 of 71). None received laboratory confirmation of the etiology because of limited medical resources.

CT examinations were performed on the NeuViz 128 CT (Neusoft, China) with automatic tube current (300 mA–496 mA), tube voltage = 120 kV. The pitch was set at 1.5 and breath-hold at full inspiration. The slice-thickness of each CT scan was 1 mm. The reconstruction matrix was 512 × 512 pixels. The image enhancement factor was 1.0. The window width was 1000, and the window level was −700.

All subjects’ demographic characteristics and clinical data were retrospectively reviewed and collected, including age, gender, exposure history, diabetes, hypertension, chronic obstructive pulmonary disease(COPD), chronic liver disease, chronic kidney disease, cancer, cardiovascular disease, fever, cough, myalgia, fatigue, headache, nausea, diarrhea, bellyache, dyspnea, other symptoms, white blood cell count, number of neutrophils, lymphocyte count, hemoglobin and platelet count. The demographic statistics of patients were summarized in Table 1. In the training cohort, COVID-19 patients had significantly older age, more exposure history, more cough, myalgia, fatigue, headache, neusea, diarrhea symptoms, lower lymphocyte count and platelet count than patients with other types of pneumonia. In both the training and testing cohort, COVID-19 patients had significantly lower white blood cell count and neutrophils than patients with other types of pneumonia.

**Table 1:** Characteristics of Patients in the training and testing Cohorts

Characteristic	Training cohort			Testing cohort		
	COVID-19	Other types of-pneumonia	P value	COVID-19	Other types of pneumonia	p value
Patients	52	60	–	11	11	–
Exams	206	69	–	38	13	–
Age	52.7 ± 12.6	41.5 ± 15.5	** < 0.001**^b^	47.5 ± 16.1	46.9 ± 18.4	0.942^b^
Gender, Male (%)	31 (60%)	33 (55%)	0.623^a^	5 (45%)	8 (73%)	0.387^a^
Exposure history			**0.006**^a^			0.375^a^
Close contact with infected patients	52 (100%)	52(87%)		8 (73%)	6 (55%)	
Unknown cause	0 (0%)3	8(13%)		3 (27%)	5 (45%)	
Comorbidities, No. (%)						
Cardiovasular disease	1 (2%)	1(2%)	1.000^a^	2 (18%)	1 (9%)	0.534^a^
Diabetes	4 (8%)	0(0%)	**0.029**^a^	0 (0%)	0 (0%)	–
Hypertension, No. (%)	9 (17%)	4(7%)	0.080^a^	0 (0%)	1 (9%)	0.317^a^
COPD	3 (6%)	2(3%)	0.535^a^	0 (0%)	0 (0%)	–
Chronic liver disease	2 (4%)	1(2%)	0.478^a^	0 (0%)	0 (0%)	–
Chronic kidney disease	0 (0%)	0(0%)	–	0 (0%)	1 (9%)	0.317^a^
Cancer, No. (%)	0 (0%)	1(2%)	0.352^a^	1 (9%)	2 (18%)	0.544^a^
Symptoms						
Fever, No. (%)	46 (88%)	46 (77%)	0.104^a^	6 (55%)	7 (64%)	0.672^a^
Cough, No. (%)	43 (83%)	26 (43%)	** < 0.001**^a^	6 (55%)	3 (27%)	0.387^a^
Myalgia, No. (%)	10 (19%)	2 (3%)	**0.007**^a^	2 (18%)	0 (0%)	0.476^a^
Fatigue, No. (%)	18 (35%)	2 (3%)	**< 0.001**^a^	0 (0%)	0 (0%)	–
Headache, No. (%)	9 (17%)	1 (2%)	**0.01**^a^	2 (18%)	0 (0%)	0.476^a^
Nausea, No. (%)	5 (10%)	0 (0%)	**0.046**^a^	2 (18%)	0 (0%)	0.476^a^
Diarrhea, No. (%)	7 (13%)	1 (2%)	**0.04**^a^	1 (9%)	0 (0%)	1.000^a^
Bellyache, No. (%)	0 (0%)	0 (0%)	–	1 (9%)	0 (0%)	1.000^a^
Dyspnea, No. (%)	1 (2%)	0 (0%)	0.464^a^	0 (0%)	0 (0%)	–
Other symptoms, No. (%)	19 (37%)	14 (23%)	0.126^a^	4 (36%)	2 (18%)	0.635^a^
Laboratory results						
White blood cell count	4.8 ± 2.0	8.2 ± 3.5	** < 0.001**^b^	5.1 ± 1.8	9.4 ± 3.4	**0.001**^b^
Number of neutrophils, × 10^9^/L	3.2 ± 1.9	5.8 ± 3.2	** < 0.001**^b^	3.2 ± 1.5	6.8 ± 3.3	**0.003**^b^
Lymphocyte count, × 10^9^/L	1.2 ± 0.8	1.6 ± 1.1	**0.032**^b^	1.4 ± 0.5	1.7 ± 1.0	0.473^b^
Hemoglobin	134.1 ± 25.2	138.7 ± 17.6	0.267^b^	128.3 ± 19.8	139.7 ± 21.3	0.206^b^
Platelet count, × 10^9^/L	175.8 ± 60.3	225.6 ± 62.0	** < 0.001**^b^	215.6 ± 70.7	230.1 ± 89.0	0.678^b^

p^a^ chi-square test, p^b^ Student’s t test. p^c^ Kruskal–Wallis H test

**Fig. 2:**
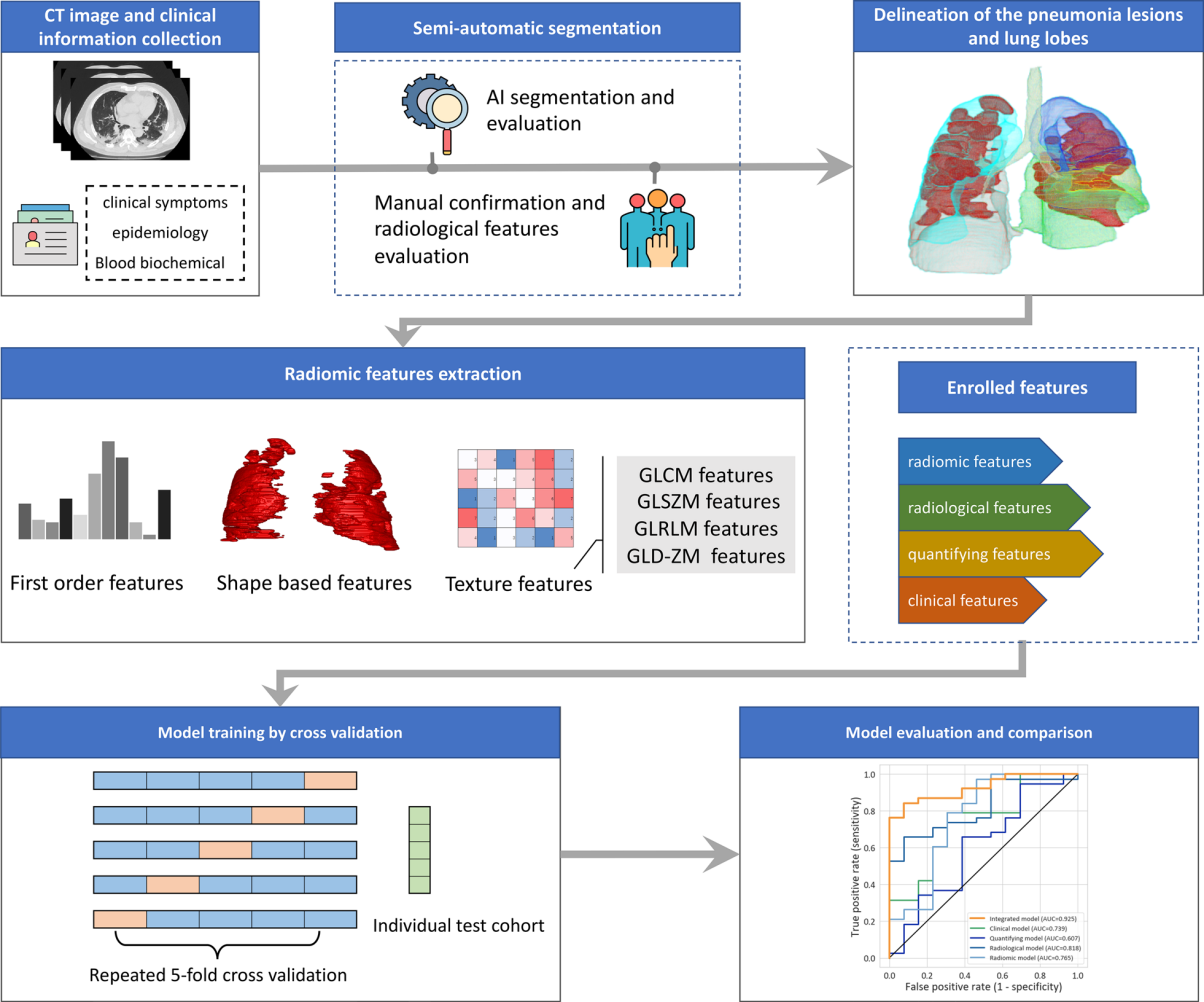
The workflow of our study, consisting of data collection, semi-automatic VOI segmentation and radiological features annotation, radiomic and quantifying features extraction, model building and evaluation

The flow chart of data collection, ROI and features annotation, radiomics, and quantity feature extraction, model building and evaluation were shown in Fig. 2.

### Lesion segmentation and radiological evaluation

All the CT scans were split into a training and a testing cohort with a ratio of 85:15 at the patient level according to the visiting time of the hospital. Feature selection and model building were performed on the training cohort, and the testing cohort was not used for the training procedure.

The pneumonia lesions were segmented semi-automatically. Firstly, the anonymized thin-slice DICOM format non-enhanced CT images were imported into an AI pneumonia assessment system, on which the pneumonia lesions were automatically detected and delineated. On the assessment platform, an MVP-Net (Multi-View FPN with Position-aware attention) which was trained on the NIH DeepLesion dataset and had achieved state-of-the-art performance [35], was used to detect abnormal patterns and classify them into consolidation and ground-glass opacity. Then a 3D U-Net model trained with a local dataset of over 10,000 lung CT scans was used to segment detected consolidation and ground-glass opacity lesions. Besides, pulmonary lobes were segmented by a pre-trained lobe segmentation model [36, 37]. Subsequently, fifteen radiologists with more than 5 years of experience in chest imaging, blind to the knowledge of the pathological report and other clinical information, refined the segmentation results (Volume of Interest, VOI) and evaluated the radiological characteristics. Each series was refined and evaluated by one of the fifteen radiologists. The segmentations and radiological characteristics were confirmed by two radiologists (F. C and Y.C) with 16 and more than 30 years of experience, respectively.

**Fig. 3:**
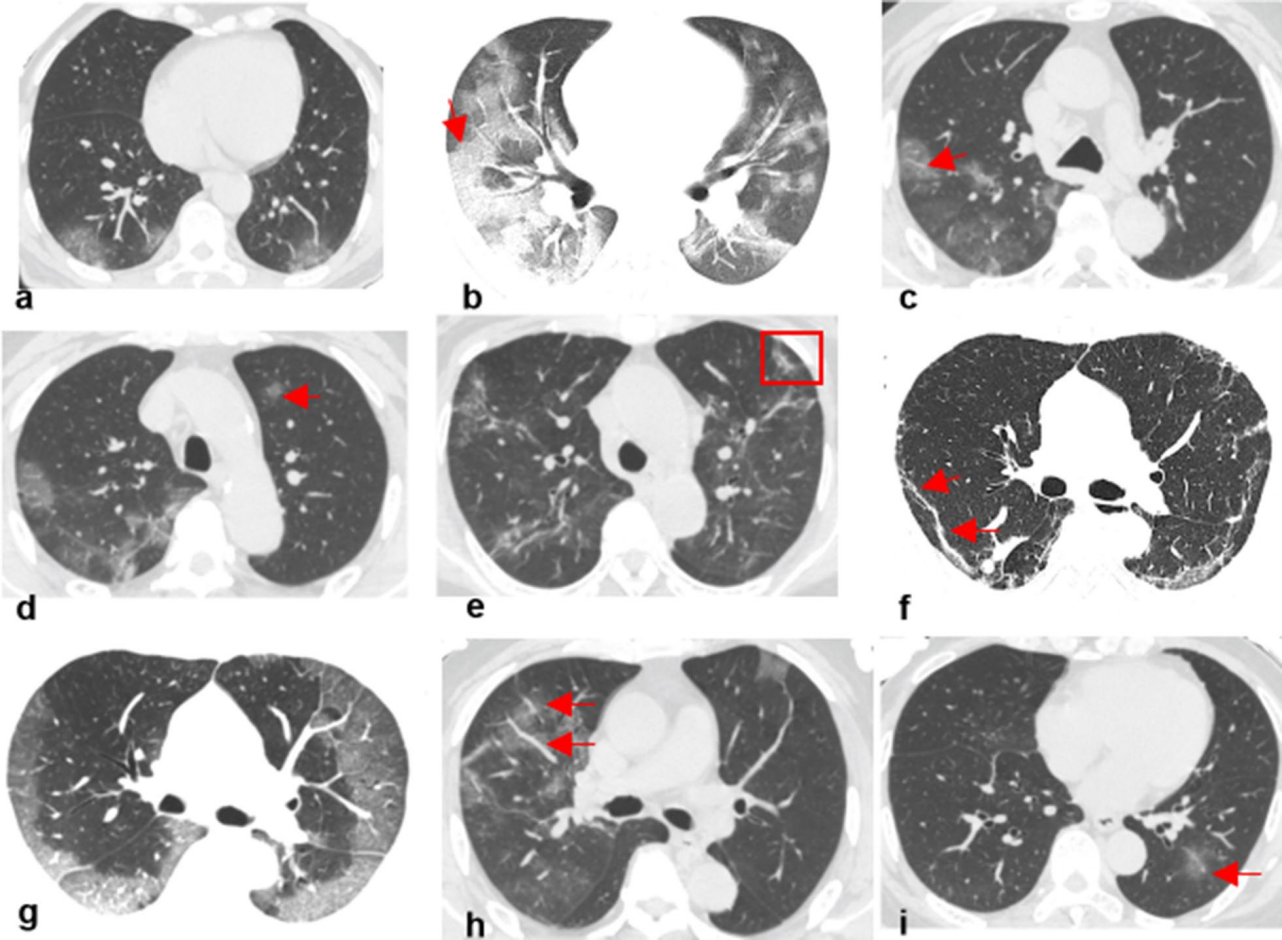
Typical radiological characteristics of CT manifestations. **a**–**i** demonstrated the typical radiological characteristics of ground grass opacity, crazy paving pattern, halo sign, reversed halo sign, vascular perforating in the lesion, subpleural line, subpleural distribution, broncho vascular bundle distribution, and pulmonary band distribution, respectively

The 7 radiological characteristics included ground-glass opacity, crazy paving pattern, halo sign, reversed halo sign, vascular perforating in the lesion, subpleural line, and lesion locations (Fig. 3). For each series, the frequency of the radiological characteristics occurring was used for modeling.

### Quantifying CT characteristics and radiomics features

The segmentation results were used to extract quantifying CT characteristics and radiomics features.

There was a total of 33 quantitative characteristics. Apart from the segmentation results, the AI pneumonia assessment system also provided the number of lesions that suffered bulla, emphysema, pleural thickening, reticular, and stripe, which were included as quantitative characteristics. Similar to the previous study [38], the mean and standard deviation of the CT values of the consolidation lesions, ground-glass lesions, and both types of lesions were calculated from the segmentation. In addition, the volumes of the consolidation lesions, ground-glass lesions, their sum, and moreover, their ratios were calculated, including the volumes of the consolidation lesions versus the volumes of the entire pulmonary and the five pulmonary lobes respectively, the ground-glass lesions versus the volumes of the entire pulmonary and the five pulmonary lobes respectively, and the volumes of both types of lesions versus the volumes of the entire pulmonary and the five pulmonary lobes, respectively.

Before radiomics features were extracted, the intensities were discretized by a fixed bin width of 25, the pixel spacing of images was resampled to 1.0 mm × 1.0 mm × 1.0 mm per pixel by the BSpline algorithm. Apart from the original images, the wavelet filters or Laplacian of Gaussian filters were performed to generate several filtered images. A total of 1218 radiomics features were extracted from the manual confirmed 3D VOIs of the original images and the filtered images by PyRadiomics V2.1.0 [39], including (1) 252 First-order features; (2) 14 Shape-based features; (3) 308 Gray Level Co-occurrence Matrix (GLCM) Features; (4) 224 Gray Level Size Zone Matrix (GLSZM) Features; (5) 224 Gray Level Run Length Matrix (GLRLM) Features; (6) 196 Gray Level Dependence Matrix (GLD-ZM) Features. The pre-processing methods and radiomic feature descriptions are detailed in Additional file 1: Information 1.1. and 1.2).

### Development of predictive models

4 groups of features were included in the model building: radiomics features, radiological features, quantity features, and clinical features. The Support Vector Machine (SVM) models with the radial basis function kernel were built on the 4 groups of features individually and on the combination of them.

Before model building, all numerical features were normalized by the z-score method, and the categorical features were encoded by the one-hot encoder. To avoid overfitting, feature selection methods were used to reduce the number of features. The optimal parameters of the combination of the feature selection methods and the model were found by grid searching with a ten-run fivefold cross-validation procedure on the training cohort. After they were determined, the model was built using the entire training cohort and the performance on the testing cohort was evaluated. After the cross-validation procedure, the threshold that maximized the Youden Index on the validation cohort was used to cut off the discriminative score to differentiate the COVID-19 from other pneumonia.

Features were selected by a two-step method. (1)The Mann–Whitney U test was used and p values were corrected by the Benjamini–Hochberg method. The features that were significantly different (p < 0.05) between the COVID-19 cohort and non-COVID-19 cohort were preserved. (2) the minimum-redundancy maximum-relevancy(mRMR) method was used and the number of selected features was determined by the cross-validation procedure. Especially, for the radiological features, the mRMR procedure was removed because there were only 7 radiological features.

The discrimination performance of the model was evaluated by the area under the receiver operator characteristic curve (AUC), accuracy (ACC), sensitivity, and specificity. The AUCs of the SVM model that built on the combined features and those on each individual feature group were compared by the Delong test. Because the SVM model with radial basis function kernel is nonlinear, the feature importance cannot be derived directly. The permutation importance [40] was used to evaluate the feature importance and the AUC was used to measure the difference between the baseline and the model that was built with the permutated feature. The consistency of the traditional radiological features was evaluated by the Kappa coefficient, and the dice coefficient between the corrected segmentation and AI segmentation results were used to evaluate the reproducibility of the radiomic features. These statistical analyses were performed on R software (version 3.6.0; https://www.r-project.org/) environments. Feature selection and model building procedures were performed by the scikit-learn package [41].

## Results

### Clinical data

Table 1 demonstrated the study population characteristics for the training and testing cohorts. Data related to age, exposure history, cough, myalgia, fatigue, headache, and diarrhea were significantly different between COVID-19 and other types of pneumonia in the training cohort (p < 0.05). Regarding the laboratory results, the white blood cell count and the number of neutrophils were significantly lower in the COVID-19 group than those in the negative group (p < 0.05) for both the training cohort and the testing cohort. In addition, the lymphocyte and plate count were significantly lower in the COVID-19 group than those in the other types of pneumonia group (p < 0.05).

### Evaluation of the model performance

**Fig. 4:**
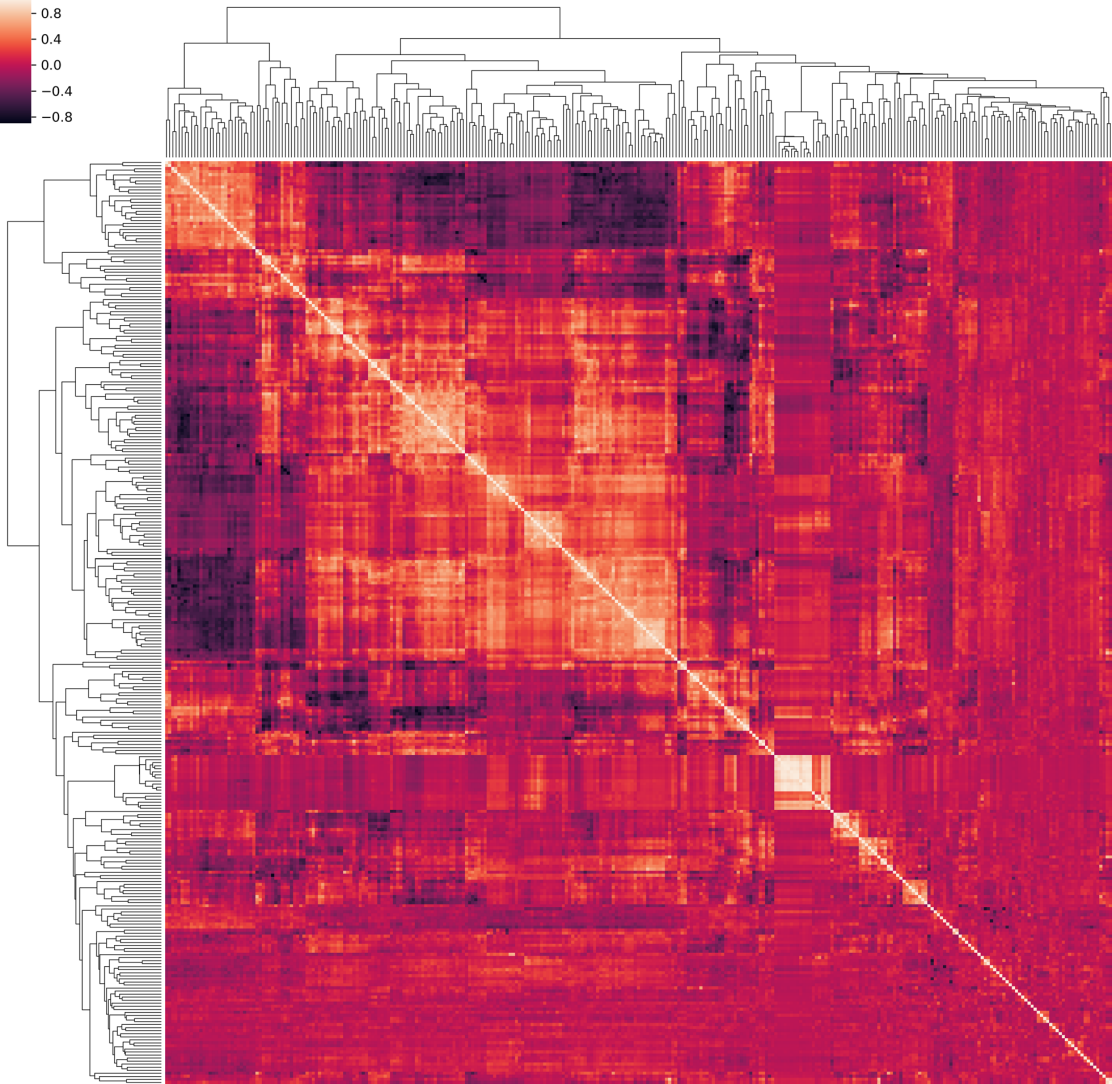
Clustered heatmap. Feature correlation matrix of radiomic features was represented as a hierarchically clustered heatmap

A total of 1128 radiomic features were extracted from each patient, the correlation cluster map was shown in Fig. 4. It can be found in the cluster map that most of the radiomic features were correlated and redundant. The dice coefficient between the corrected segmentation and the AI segmentation result reached 0.82 ± 0.14, indicating the satisfactory performance of the AI segmentation performance and the robustness of the radiomic feature extraction. For the ground-glass opacity, crazy paving pattern, halo sign, reversed halo sign, vascular perforating in the lesion, subpleural line, and lesion locations, the Kappa values were 0.728, 0.733, 0.728, 0.701, 0.841, 0.866, 0.818, respectively.

**Fig. 5:**
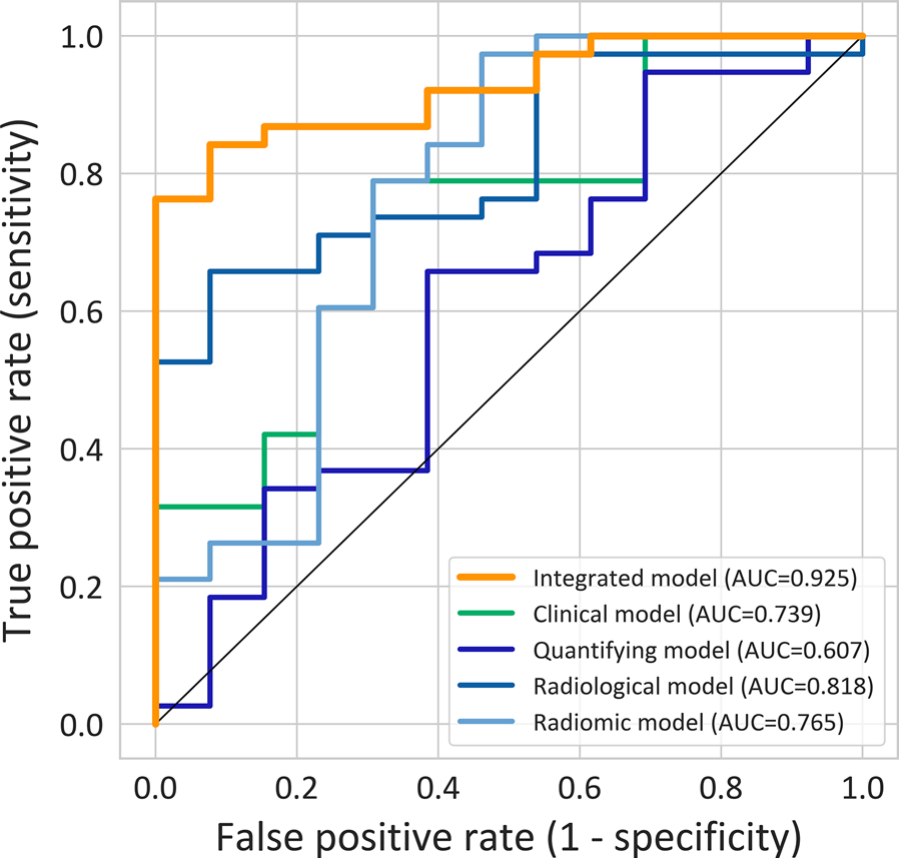
The ROC of the integrated model, clinical model, quantifying model, radiological model, and radiomic model on the testing cohort

For the SVM model that built on the combination of 4 groups of features, it reached an AUC of 0.984 (0.971 to 0.997), 0.893 (0.841 to 0.946), and 0.925 (0.856 to 0.994) on the training, cross-validation, and testing cohort. For the sensitivity and specificity, it reached 0.816 and 0.923 on the test cohort. For the SVM models that built on radiomic features, radiological features, quantifying features, and clinical features individually, the AUC on the testing cohort reached 0.765 (95% CI 0.585 to 0.946), 0.818 (95% CI 0.698 to 0.938), 0.607 (95% CI 0.414 to 0.8) and 0.739 (95% CI 0.58 to 0.898) respectively, significantly lower than the integrated model, except for the radiomic model. The details of the performance are shown in Table 2 and the ROC curve of the 4 SVM models on the time-independent test cohort was shown in Fig. 5.

**Table 2:** The performance of CT radiomics models in training, cross-validation and testing cohorts

Models	Dataset	AUC (95% CI)	ACC (95% CI)	Specificity (95% CI)	Sensitivity (95% CI)
Clinical model	Training	0.942 (0.916 to 0.969)	0.844 (0.794 to 0.883)	0.87 (0.762 to 0.935)	0.835 (0.776 to 0.882)
	Validation	0.881 (0.835 to 0.927)	0.793 (0.739 to 0.838)	0.826 (0.712 to 0.903)	0.782 (0.718 to 0.835)
	Testing	0.739 (0.58 to 0.898)^a^	0.647 (0.5 to 0.772)	0.769 (0.46 to 0.938)	0.605 (0.435 to 0.755)
Radiological model	Training	0.922 (0.89 to 0.955)	0.804 (0.751 to 0.848)	0.957 (0.87 to 0.989)	0.752 (0.687 to 0.809)
	Validation	0.869 (0.82 to 0.918)	0.775 (0.72 to 0.822)	0.899 (0.796 to 0.955)	0.733 (0.666 to 0.791)
	Testing	0.818 (0.698 to 0.938)^a^	0.588 (0.442 to 0.721)	1 (0.717 to 1)	0.447 (0.29 to 0.615)
Radiomic model	Training	0.962 (0.939 to 0.986)	0.909 (0.867 to 0.939)	0.884 (0.779 to 0.945)	0.917 (0.869 to 0.95)
	Validation	0.828 (0.767 to 0.889)	0.825 (0.774 to 0.867)	0.797 (0.68 to 0.881)	0.835 (0.776 to 0.882)
	Testing	0.765 (0.585 to 0.946)	0.667 (0.52 to 0.789)	0.692 (0.389 to 0.896)	0.658 (0.486 to 0.799)
Quantifying model	Training	0.899 (0.863 to 0.935)	0.815 (0.762 to 0.858)	0.812 (0.696 to 0.892)	0.816 (0.754 to 0.865)
	Validation	0.803 (0.742 to 0.863)	0.778 (0.724 to 0.825)	0.725 (0.602 to 0.822)	0.796 (0.733 to 0.848)
	Testing	0.607 (0.414 to 0.8)^a^	0.608 (0.461 to 0.738)	0.615 (0.323 to 0.849)	0.605 (0.435 to 0.755)
Integrated model	Training	0.984 (0.971 to 0.997)	0.956 (0.923 to 0.976)	0.899 (0.796 to 0.955)	0.976 (0.941 to 0.991)
	Validation	0.893 (0.841 to 0.946)	0.88 (0.834 to 0.915)	0.754 (0.633 to 0.846)	0.922 (0.875 to 0.954)
	Testing	0.925 (0.856 to 0.994)	0.843 (0.709 to 0.925)	0.923 (0.621 to 0.996)	0.816 (0.651 to 0.917)

^a^DeLong test showed significant different (p < 0.05) between the model with integrated model on the testing cohort. CI confidence interval

**Fig. 6:**
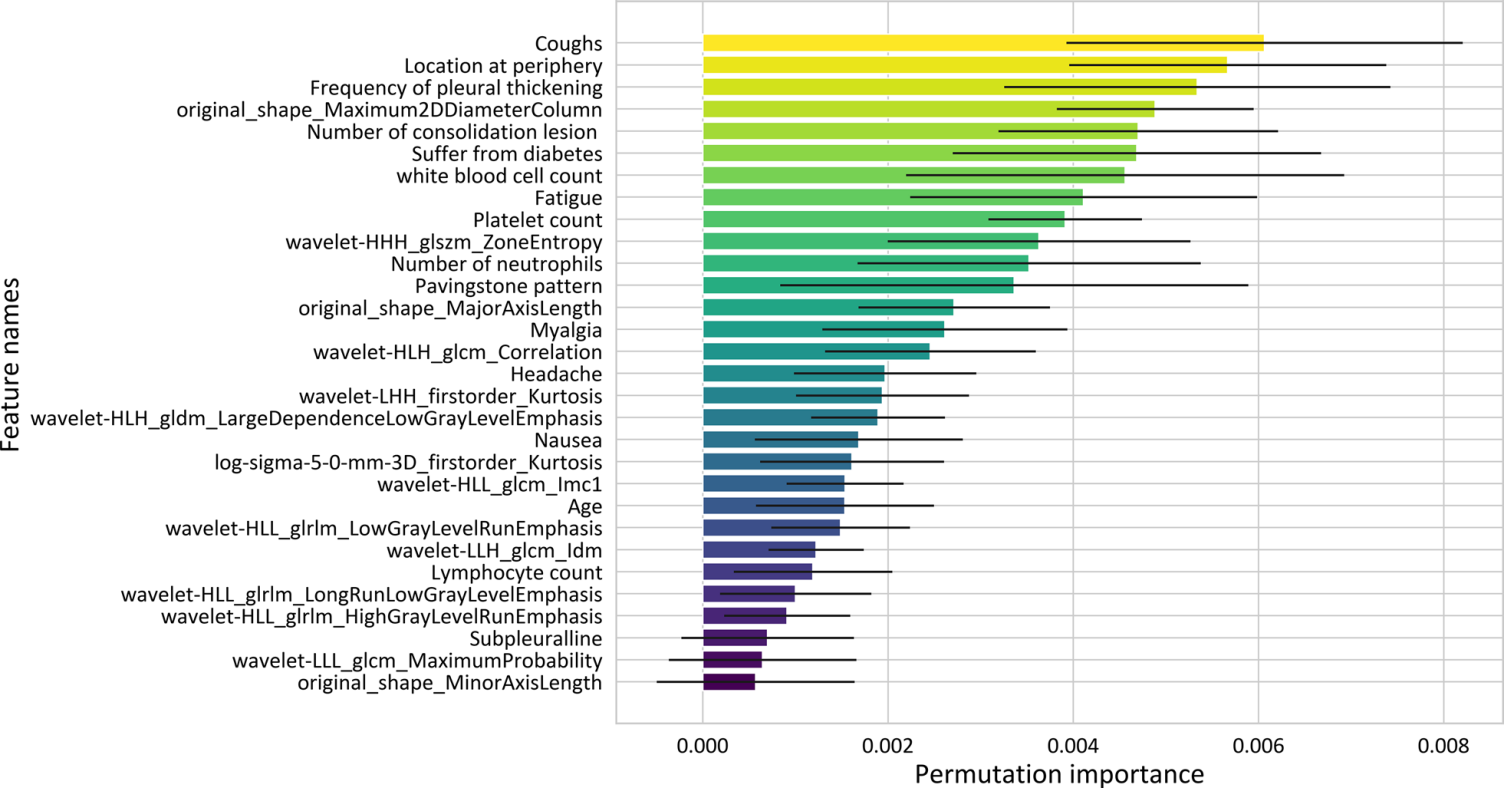
The permutation feature importance of the integrated model

There were 30 features involved in the integrated SVM model building, including 14 radiomic features, 9 clinical features, 4 quantifying features, and 3 radiological features. The feature importance of these features was shown in Fig. 6.

**Fig. 7:**
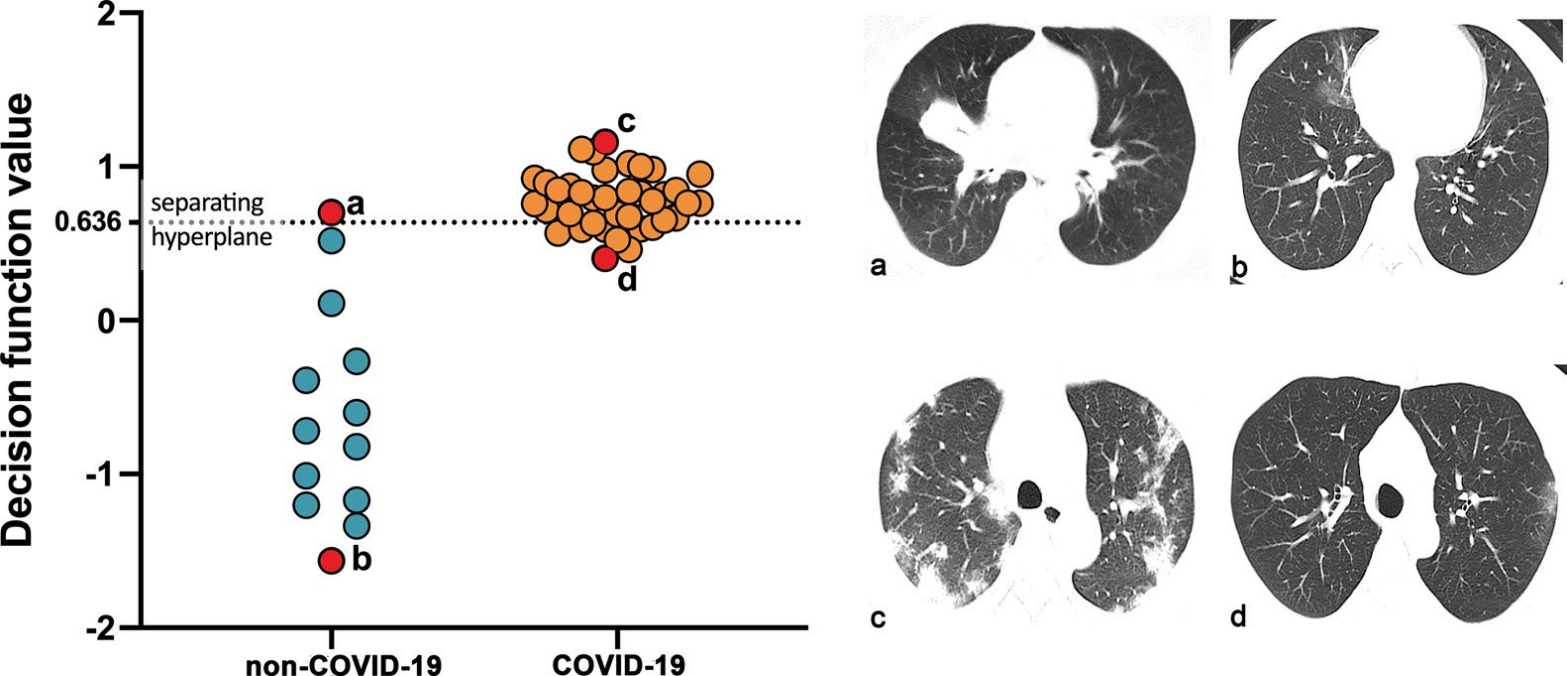
The decision function value distribution of the patients with non-COVID-19 pneumonia and COVID-19 in the test cohort was shown. Each point indicated a patient in the test cohort, the non-COVID-19 point below the adjusted separating hyperplane line and the COVID-19 point above the line were separated correctly. The images of the 4 typical patients were shown. **a** A patient with non-COVID-19 that misclassified as COVID-19. **b** A patient with non-COVID-19 that were correctly identified. **c** A patient with COVID-19 that were correctly identified. **d **A patient with COVID-19 that misclassified as non-COVID-19

Figure 7 showed the decision function value distribution of the non-COVID-19 pneumonia and COVID-19 in the test cohort. The function values were proportional to the distance of the patient to the separating hyperplane, thus indicating the integrated model’s confidence in the result of classification. The separating hyperplane was adjusted to maximize the Youden index on the cross-validation cohort. From the CT images, we could see that when the lesions of COVID-19 were at the absorption stage, they became small, and thus it was difficult to differentiate from non-COVID-19 pneumonia. On the contrary, when the lesions of COVID-19 were relatively big, it was easy to differentiate it from non-COVID-19 pneumonia with typical lesion locations and CT manifestation.

## Discussion

In this study, we developed and tested a machine learning-based CT radiomics model for classifying COVID-19 from non-COVID-19 pneumonia on chest CT images. CT radiomics features of lesions were extracted, and the model showed good performance on the training cohort, cross-validation result, and testing cohort. On the testing dataset, our result revealed that this model achieved a high sensitivity of 0.816 (95% CI 0.651 to 0.917) and a high specificity of 0.923 (95% CI 0.621 to 0.996) in diagnosing COVID-19. As far as we are concerned, this is the first study that uses comprehensive information by including both imaging and clinical data in the classification of COVID-19.

Since the outbreak of COVID-19, clinical characteristics have been regarded as important clues for diagnosing COVID-19. However, the value of clinical characteristics in the diagnosis of COVID has not yet been fully evaluated. Our present study revealed that clinical features were valuable, but not the only strong clue for diagnosing COVID-19. This result is of great significance since COVID-19 confirmed cases is still rising all over the world. We have included both COVID-19 patients without a history of exposure and non-COVID-19 patients with a history of exposure in the current study. Exposure history has been regarded as an important indicator in diagnosing COVID. Besides, our study demonstrated that when compared with non-COVID-19 patients, COVID-19 patients had significantly lower leukocyte, neutrophils, lymphocyte, and platelet counts. It could be explained that because COVID-19 belongs to viral infection, whereas non-COVID-19 patients were likely to be diagnosed as bacterial infection with high leukocyte count. This is consistent with the previous study that normal or abnormally low leukocyte and lymphocyte was found to be significant indicators for diagnosing COVID-19 [42].

CT manifestations of COVID-19 have been deemed as an indispensable role for the clinical diagnosis of COVID-19 [38]. However, few studies have elucidated the role of CT features in diagnosing COVID-19. Therefore, we have assessed the diagnostic value of radiological characteristics including ground-glass opacity, crazy paving pattern, halo sign, reversed halo sign, vascular perforating in the lesion, subpleural line, and lesion locations in our study. Among these features, those located at the periphery seemed to be the most important for the classification. This was in line with the previous study in which the lesions of COVID-19 were distributed mainly in the subpleural area [43]. We found that when only the radiological features were included, the model revealed a good performance of AUCs for training, validation, and testing cohort, 92.2%, 86.9% and 81.8%, respectively. This result was in accord with the previous study [38], in which the model was built on the basis of the clinical data, laboratory results, and CT features. Our study indicated that CT is valuable for diagnosing COVID-19.

The encouraging diagnostic performance of the machine learning-based CT radiomics model indicates that radiomics might be particularly helpful for the detection of COVID-19 as the AUCs of other models in the testing dataset were significantly lower than that of the integrated model, except for the radiomics model. Radiomics features in our model included first-order features, shape-based features, and the distribution, correlation, and variance in gray level intensities. These radiomics features described the relationship between voxels and contained quantitative information on the spatial heterogeneity of pneumonia lesions. Importantly, when only including radiomics features, the model revealed the good performance of AUCs for training, validation, and testing cohort, 96.2%, 82.8% and 76.5%, respectively. Similarly, Fang et al. found that the radiomics model has outperformed the clinical model in the prediction/diagnosis of COVID-19 pneumonia [30]. By using deep learning classifier multi-layer perceptron (DL-MLP), Zhang et al. found that DL-MLP achieved optimal performance with AUC of 0.922 (95% CI 0.856–0.988) and 0.959 (95% CI 0.910–1.000), the same sensitivity of 0.879, and specificity of 0.900 and 0.887 on internal and external testing datasets, indicating that DL-MLP may be helpful in efficiently screening COVID-19 patients [29]. Besides, Tan et al. demonstrated that automatic machine learning based on radiomics of non‑focus area in the first chest CT could be used to distinguish different clinical types of COVID‑19 [31]. To summarize, radiomics was useful in controlling the spread of COVID-19. Importantly, by combining the radiological features, quantifying features, and clinical characteristics, the performance of the model was significantly improved. Its AUCs on training, validation, and testing cohorts were all over 89%, indicating that the models have the potential to be applied in a general situation. By using deep learning techniques, a previous study was able to distinguish COVID-19 from community-acquired pneumonia [11]. We were able to collect several patients with other types of pneumonia diagnosis on CT of the corresponding period. More importantly, these types of pneumonia were highly suspected of COVID-19 in consideration of the epidemic, CT findings, and laboratory results.

A majority of the countries all over the world have been affected by COVID-19. Early diagnosis is of importance for preventing the spread of the disease. Though RT-PCR is considered as the gold standard for the diagnosis of COVID-19, CT is used as an effective supplementary tool for the diagnosis of COVID-19 [8, 9]. Our study revealed that the machine learning-based CT radiomics model by combining radiomics, subjective characteristics, quantitative characteristics, and clinical characteristics achieved good performance for the diagnosis of COVID-19 and differentiating it from non-COVID-19 pneumonia. This is in line with the idea that adding additional clinical information could significantly improve the performance of radiomics [44, 45]. Shiri et al. revealed that the combination of radiomic features, clinical and radiological data could effectively predict survival in COVID-19 patients [44]. Similarly, Chao et al. demonstrated that the integration of both imaging and non-imaging data significantly improved the performance of prediction to need for ICU admission in patients with COVID-19 pneumonia [45]. All in all, holistic information is effective in the diagnosis of COVID-19.

The study has several limitations. First, the sample size was relatively small. A larger prospective multicenter cohort is needed to test the effectiveness of machine learning-based CT radiomics models. Second, patients with non-COVID-19 pneumonia did not receive laboratory confirmation of the etiology because of limited medical resources during the COVID-19 outbreak. Thirdly, we did not use quantitative characteristics to evaluate the evolution of the disease. Future work should include quantitative information regarding disease progression. Regarding the field of radiomics, it remains unclear which algorithm, classifiers, and feature selector would achieve optimal results for investigation [46–48]. In the present study, we integrated different biological and clinical information together with radiomics, and better diagnostic performance was achieved. This was in line with the study of Parmar et al. [49], who found that a comparative investigation could be helpful in the identification of the optimal and reliable machine learning methods for radiomics-based prognostic analyses. Future studies should integrate different biological and clinical information together with radiomics.

## Conclusions

In conclusion, a machine learning-based CT radiomics model is valuable for accurately classifying COVID-19, which would be helpful for clinicians and radiologists to identify COVID-19 patients.

## Supplementary Information

**Additional file 1.** The pre-processing methods and radiomic feature descriptions are detailed.

**Additional file 2.** For the clinical model, the feature importance was shown. The top 3 important clinical factors were the occurrence of fatigue, age and the occurrence of cough.

**Additional file 3.** The feature importance of the quantifying model was shown. The frequency of the pleural thickening, consolidation lesion and ground glass lesion were the top 3 importance features.

**Additional file 4.** The radiological model was shown. The frequency occurrence of paving stone, position at periphery, and subpleural line were importance for the discrimination of the COVID-19 from other pneumonia.

**Additional file 5.** The feature importance of the radiomic model was shown. The most important feature was Zone Entropy of glszm on the wavelet filtered image, indicating the heterogeneneity in the texture patterns. The shape of the lesion was also important, and the Minor Axis Length and Maximum 2D Diameter Slice were the second and third most important radiomic features.

## Data Availability

The datasets used during the current study are available from the corresponding author on reasonable request.

## Abbreviations

COVID-19: Coronavirus disease 2019
ROI: Region of interest
SVM: Support Vector Machine
WHO: World Health Organization
PHEIC: Public health emergency of international concern
RT-PCR: Reverse-transcription polymerase chain reaction
CT: Computed tomography
GLSZM: Gray Level Size Zone Matrix Features
GLRLM: Gray Level Run Length Matrix Features
GLD-ZM: Gray Level Dependence Matrix Features
AUC: Area under the receiver operator characteristic curve
