# Supplementary material for: Machine learning-based CT radiomics model distinguishes COVID-19 from non-COVID-19 pneumonia
Source: BMC Infect Dis. 2021 Sep 8;21:931. doi: 10.1186/s12879-021-06614-6 (PMC8424152; doi:10.1186/s12879-021-06614-6)
Supplement: Supplementary file 1 — Additional file 1. The pre-processing methods and radiomic feature descriptions are detailed. [file 12879_2021_6614_MOESM1_ESM.docx]

1. **Extraction of radiomic features**
   1. **Pre-processing methods**

In this study, two filtering processes were performed to give additional first-order and textural characteristics. For the wavelet filter, a discrete, one-level and “Coiflet 1” three dimensional wavelet transformation was applied to each CT image, which decomposed the original image X into 8 decompositions. Consider H and L to be a high-pass function and low-pass function respectively, and the decomposition of X can be labeled as $X_{LLL}$, $X_{LLH}$,$X_{LHL}$,$X_{LHH}$,$X_{HLL}$,$X_{HLH}$, $X_{HHL}$ and$X_{HHH}$. The subscripts denoted the type of filters that were applied along x, y, and z direction.

The LoG filter was consisted of a proceeding Gaussian filter and a Laplacian filter. The Gaussian kernel is used to smooth the image and reduce noise and the Laplacian operator is sensitive to areas with rapidly changing intensities. The LoG was often used to intensify the edge information of intrested area. The Gaussian kernel is defined as

$$G\left( x,y,z,\sigma\right)= \frac{1}{{(\sigma\sqrt{2\pi})}^{3}}e^{-\frac{x^{2}+y^{2}+z^{2}}{2\sigma^{2}}}$$

which is then convolved by the laplacian kernel $\nabla^{2}G(x,y,z)$. The width of the filter in the Gaussian kernel is determined by σ and was set at 1, 2, 3, 4, and 5 in this study. Lowere value is associated with more fine texture and higher value is associated with more coarse textures.

- 1. **Radiomic features**

Radiomic features were divided into six groups according to different types: (1) first-order features, (2) shape-based features, (3) gray level co-occurrence matrix (GLCM) features, (4) gray level size zone matrix (GLSZM) features, (5) gray level run-length matrix (GLRLM) features and (6) gray level dependency matrix (GLDM) features. GLCM, GLSZM, GLRLM and GLDM are essentially all textural features. **The description of the features was obtained by reorganizing the information in the documentation of the PyRadiomics software (https://pyradiomics.readthedocs.io/en/latest/features.html), and for formulas of each feature, please refer to the documentation.**

1. First-order features, also known as histogram features, include 18 features, which describe the distribution of voxel intensties within the VOI.
2. Shape features include 4 features, which describe the morphological property of the tumor’s outline.
3. GLCM features include 22 features, which describe the the spatial dependence of each pixel in the tumor region.
4. GLSZM features include 16 features, which quantify the gray level zone in the tumor region. It is an advanced statistical matrix for textural feature characterization.
5. GLRLM features include 16 features, which quantify gray level runs and describe the textural characteristics of the tumor region .
6. GLDM features include 14 features, which describe the complex texutral characteristics in the tumor region. This algorithm is insensitive to monotonic gray level transformation.

First-order features and textural features were obtained from original CT images, 8 wavelet filtered images and 5 LoG filtered images. Shape-based features were only obtained from the original images for that filtered images no longer reflect the true morphology of tumors. For each VOI, a total of 1218 quantitative imaging features were extracted. Among them, there were 252 (18 types of features $\times$14 types of images) first-order features, 14 were shape based features, 308 (22 types of features $\times$14 types of images) GLCM features, 224 (16 types of features $\times$14 types of images) GLSZM features, 224 (16 types of features $\times$14 types of images) GLRLM features and 196 (14 types of features $\times$14 types of images) GLDM features. The features in each group were detailed in Table S1 and listed below.

Table S1. The summary of the feature extraction.

| Type | image that extracted from | number of features |
| --- | --- | --- |
| firstorder | Log filter with sigma of 1mm | 18 |
| firstorder | Log filter with sigma of 2mm | 18 |
| firstorder | Log filter with sigma of 3mm | 18 |
| firstorder | Log filter with sigma of 4mm | 18 |
| firstorder | Log filter with sigma of 5mm | 18 |
| firstorder | original | 18 |
| firstorder | wavelet filter with HHH pass for xyz orientation | 18 |
| firstorder | wavelet filter with HHL pass for xyz orientation | 18 |
| firstorder | wavelet filter with HLH pass for xyz orientation | 18 |
| firstorder | wavelet filter with HLL pass for xyz orientation | 18 |
| firstorder | wavelet filter with LHH pass for xyz orientation | 18 |
| firstorder | wavelet filter with LHL pass for xyz orientation | 18 |
| firstorder | wavelet filter with LLH pass for xyz orientation | 18 |
| firstorder | wavelet filter with LLL pass for xyz orientation | 18 |
| glcm | Log filter with sigma of 1mm | 22 |
| glcm | Log filter with sigma of 2mm | 22 |
| glcm | Log filter with sigma of 3mm | 22 |
| glcm | Log filter with sigma of 4mm | 22 |
| glcm | Log filter with sigma of 5mm | 22 |
| glcm | original | 22 |
| glcm | wavelet filter with HHH pass for xyz orientation | 22 |
| glcm | wavelet filter with HHL pass for xyz orientation | 22 |
| glcm | wavelet filter with HLH pass for xyz orientation | 22 |
| glcm | wavelet filter with HLL pass for xyz orientation | 22 |
| glcm | wavelet filter with LHH pass for xyz orientation | 22 |
| glcm | wavelet filter with LHL pass for xyz orientation | 22 |
| glcm | wavelet filter with LLH pass for xyz orientation | 22 |
| glcm | wavelet filter with LLL pass for xyz orientation | 22 |
| gldm | Log filter with sigma of 1mm | 14 |
| gldm | Log filter with sigma of 2mm | 14 |
| gldm | Log filter with sigma of 3mm | 14 |
| gldm | Log filter with sigma of 4mm | 14 |
| gldm | Log filter with sigma of 5mm | 14 |
| gldm | original | 14 |
| gldm | wavelet filter with HHH pass for xyz orientation | 14 |
| gldm | wavelet filter with HHL pass for xyz orientation | 14 |
| gldm | wavelet filter with HLH pass for xyz orientation | 14 |
| gldm | wavelet filter with HLL pass for xyz orientation | 14 |
| gldm | wavelet filter with LHH pass for xyz orientation | 14 |
| gldm | wavelet filter with LHL pass for xyz orientation | 14 |
| gldm | wavelet filter with LLH pass for xyz orientation | 14 |
| gldm | wavelet filter with LLL pass for xyz orientation | 14 |
| glrlm | Log filter with sigma of 1mm | 16 |
| glrlm | Log filter with sigma of 2mm | 16 |
| glrlm | Log filter with sigma of 3mm | 16 |
| glrlm | Log filter with sigma of 4mm | 16 |
| glrlm | Log filter with sigma of 5mm | 16 |
| glrlm | original | 16 |
| glrlm | wavelet filter with HHH pass for xyz orientation | 16 |
| glrlm | wavelet filter with HHL pass for xyz orientation | 16 |
| glrlm | wavelet filter with HLH pass for xyz orientation | 16 |
| glrlm | wavelet filter with HLL pass for xyz orientation | 16 |
| glrlm | wavelet filter with LHH pass for xyz orientation | 16 |
| glrlm | wavelet filter with LHL pass for xyz orientation | 16 |
| glrlm | wavelet filter with LLH pass for xyz orientation | 16 |
| glrlm | wavelet filter with LLL pass for xyz orientation | 16 |
| glszm | Log filter with sigma of 1mm | 16 |
| glszm | Log filter with sigma of 2mm | 16 |
| glszm | Log filter with sigma of 3mm | 16 |
| glszm | Log filter with sigma of 4mm | 16 |
| glszm | Log filter with sigma of 5mm | 16 |
| glszm | original | 16 |
| glszm | wavelet filter with HHH pass for xyz orientation | 16 |
| glszm | wavelet filter with HHL pass for xyz orientation | 16 |
| glszm | wavelet filter with HLH pass for xyz orientation | 16 |
| glszm | wavelet filter with HLL pass for xyz orientation | 16 |
| glszm | wavelet filter with LHH pass for xyz orientation | 16 |
| glszm | wavelet filter with LHL pass for xyz orientation | 16 |
| glszm | wavelet filter with LLH pass for xyz orientation | 16 |
| glszm | wavelet filter with LLL pass for xyz orientation | 16 |
| shape | original | 14 |

***Group 1. First-order features***

First-order statistics describe the distribution of voxel intensities within the CT image region defined by the mask through commonly used and basic metrics. X is a set of Np voxels included in the VOI.

**Table S2.** Descriptions of all features for first-order characteristics.

| No. | Feature names | Descriptions |
| --- | --- | --- |
| 1 | 10 Percentile | The 10th percentile of X |
| 2 | 90 Percentile | The 90th percentile of X |
| 3 | Energy | Energy is a measure of the magnitude of voxel values in an image. A larger value implies a greater sum of the squares of these values. |
| 4 | Entropy | Entropy specifies the uncertainty/randomness in the image values. It measures the average amount of information required to encode the image values. |
| 5 | Interquartile Range | Here P25 and P75 are the 25^th^ and 75^th^ percentile of the image array, respectively. |
| 6 | Kurtosis | Kurtosis is a measure of the ‘peakedness’ of the distribution of values in the image VOI. A higher kurtosis implies that the mass of the distribution is concentrated towards the tail(s) rather than towards the mean. A lower kurtosis implies the reverse: that the mass of the distribution is concentrated towards a spike near the Mean value. |
| 7 | Maximum | The maximum gray level intensity within the VOI. |
| 8 | Mean Absolute Deviation | Mean Absolute Deviation is the mean distance of all intensity values from the Mean Value of the image array. |
| 9 | Mean | The average gray level intensity within the VOI. |
| 10 | Median | The median gray level intensity within the VOI. |
| 11 | Minimum | The minimum gray level intensity within the VOI. |
| 12 | Range | The range of gray values in the VOI. |
| 13 | Robust Mean Absolute Deviation | Robust Mean Absolute Deviation is the mean distance of all intensity values from the Mean Value calculated on the subset of image array with gray levels in between, or equal to the 10th and 90th percentile. |
| 14 | Root Mean Squared | RMS is the square-root of the mean of all the squared intensity values. It is another measure of the magnitude of the image values. This feature is volume-confounded, a larger value of *c* increases the effect of volume-confounding. |
| 15 | Skewness | Skewness measures the asymmetry of the distribution of values about the Mean value. Depending on where the tail is elongated and the mass of the distribution is concentrated, this value can be positive or negative. |
| 16 | Total Energy | Total Energy is the value of Energy feature scaled by the volume of the voxel in cubic mm. |
| 17 | Uniformity | Uniformity is a measure of the sum of the squares of each intensity value. This is a measure of the homogeneity of the image array, where a greater uniformity implies a greater homogeneity or a smaller range of discrete intensity values. |
| 18 | Variance | Variance is the mean of the squared distances of each intensity value from the Mean value. This is a measure of the spread of the distribution about the mean. |

***Group 2. Shape features***

Shape features describe the morphological property of the VOI and were generated from only the image without filtration. $N_{v}$ represent the number of voxels included in the VOI. $N_{f}$ represent the number of faces (triangles) defining the Mesh.

**Table S3** Descriptions of all features for shape-based characteristics.

| No. | Feature names | Descriptions |
| --- | --- | --- |
| 1 | Elongation | Elongation shows the relationship between the two largest principal components in the VOI shape. |
| 2 | Flatness | Flatness shows the relationship between the largest and smallest principal components in the VOI shape. |
| 3 | Least Axis Length | This feature yields the smallest axis length of the VOI-enclosing ellipsoid and is calculated using the largest principal component λ*_least_*. |
| 4 | Major Axis Length | This feature yields the largest axis length of the VOI-enclosing ellipsoid and is calculated using the largest principal component λ*_major_*. |
| 5 | Maximum 2D Diameter Column | Maximum 2D diameter (Column) is defined as the largest pairwise Euclidean distance between tumor surface mesh vertices in the row-slice (usually the coronal) plane. |
| 6 | Maximum 2D Diameter Row | Maximum 2D diameter (Row) is defined as the largest pairwise Euclidean distance between tumor surface mesh vertices in the column-slice (usually the sagittal) plane. |
| 7 | Maximum2D Diameter Slice | Maximum 2D diameter (Slice) is defined as the largest pairwise Euclidean distance between tumor surface mesh vertices in the row-column (generally the axial) plane. |
| 8 | Maximum 3D Diameter | Maximum 3D diameter is defined as the largest pairwise Euclidean distance between tumor surface mesh vertices. |
| 9 | Mesh Volume | The volume of the VOI V is calculated from the triangle mesh of the VOI. For each face ii in the mesh, defined by points $a_{i}$,$b_{i}$, and $c_{i}$, the (signed) volume $V_{f}$ of the tetrahedron defined by that face and the origin of the image (O) is calculated. The sign of the volume is determined by the sign of the normal, which must be consistently defined as either facing outward or inward of the VOI. |
| 10 | Minor Axis Length | This feature yields the second-largest axis length of the VOI-enclosing ellipsoid and is calculated using the largest principal component λ*_mino_*_r_. |
| 11 | Sphericity | Sphericity is a measure of the roundness of the shape of the tumor region relative to a sphere. It is a dimensionless measure, independent of scale and orientation. The value range is 0<sphericity≤10<sphericity≤1, where a value of 1 indicates a perfect sphere (a sphere has the smallest possible surface area for a given volume, compared to other solids). |
| 12 | Surface Area | To calculate the surface area, first the surface area Ai of each triangle in the mesh is calculated. The total surface area is then obtained by taking the sum of all calculated sub-areas. |
| 13 | Surface Volume Ratio | A lower value indicates a more compact (sphere-like) shape. This feature is not dimensionless, and is therefore (partly) dependent on the volume of the VOI. |
| 14 | Voxel Volume | The volume of the VOI is approximated by multiplying the number of voxels in the VOI by the volume of a single voxel. This is a less precise approximation of the volume and is not used in subsequent features. This feature does not make use of the mesh and is not used in calculation of other shape features. |

***Group 3. Gray-Level Co-Occurrence Matrix (GLCM) features***

A GLCM is defined as $P(i,j;\delta,\alpha)$, a matrix with size $N_{g}\times N_{g}$ describing the second-order joint probability function of an image, where the $(i,j)$th element represents the number of times the combination of intensity levels $i$ and $j$ occur in two pixels in the image, that are separated by a distance of $\delta$ pixels in direction $\alpha$, and $N_{g}$ is the number of discrete gray level intensities. In this study, distance $\delta$ was set to 1 and direction $\alpha$ to each of the 13 directions in three dimensions, yielding a total of 13 gray level co-occurrence matrices for each 3D image. From these gray-level co-occurrence matrices, several textural features are derived. Each 3D gray level co-occurrence based feature was then calculated as the mean of the feature calculations for each of the 13 directions.

**Table S4.** Descriptions of all features for GLCM characteristics.

| No. | Feature names | Descriptions |
| --- | --- | --- |
| 1 | Auto Correlation | Auto correlation is a measure of the magnitude of the fineness and coarseness of texture. |
| 2 | Joint Average | The mean gray level intensity of the $i$ distribution. |
| 3 | Cluster Prominence | Cluster Prominence is a measure of the skewness and asymmetry of the GLCM. A higher value implies more asymmetry about the mean while a lower value indicates a peak near the mean value and less variation about the mean. |
| 4 | Cluster Shade | Cluster Shade is a measure of the skewness and uniformity of the GLCM. A higher cluster shade implies greater asymmetry about the mean. |
| 5 | Cluster Tendency | Cluster Tendency is a measure of groupings of voxels with similar gray-level values. |
| 6 | Contrast | Contrast is a measure of the local intensity variation, favoring values away from the diagonal (i=j). A larger value correlates with a greater disparity in intensity values among neighboring voxels. |
| 7 | Correlation | Correlation is a value between 0 (uncorrelated) and 1 (perfectly correlated) showing the linear dependency of gray level values to their respective voxels in the GLCM. |
| 8 | Difference Average | Difference Average measures the relationship between occurrences of pairs with similar intensity values and occurrences of pairs with differing intensity values. |
| 9 | Difference Entropy | Difference Entropy is a measure of the randomness/variability in neighborhood intensity value differences. |
| 10 | Difference Variance | Difference Variance is a measure of heterogeneity that places higher weights on differing intensity level pairs that deviate more from the mean. |
| 11 | Joint Energy | Energy is a measure of homogeneous patterns in the image. A greater Energy implies that there are more instances of intensity value pairs in the image that neighbor each other at higher frequencies. |
| 12 | Joint Entropy | Joint entropy is a measure of the randomness/variability in neighborhood intensity values. |
| 13 | Informational Measure of Correlation (IMC1) | IMC1 assesses the correlation between the probability distributions of i and j, quantifying the complexity of the texture. |
| 14 | Informational Measure of Correlation (IMC2) | IMC2 also assesses the correlation between the probability distributions of i and j, quantifying the complexity of the texture. |
| 15 | Inverse Difference Moment (IDM) | IDM (a.k.a Homogeneity 2) is a measure of the local homogeneity of an image. |
| 16 | Inverse Difference Moment Normalized (IDM) | IDMN (inverse difference moment normalized) is a measure of the local homogeneity of an image. |
| 17 | Inverse Difference (Id) | ID (a.k.a. Homogeneity 1) is another measure of the local homogeneity of an image. With more uniform gray levels, the denominator will remain low, resulting in a higher overall value. |
| 18 | Inverse Difference Normalized (IDN) | IDN (inverse difference normalized) is another measure of the local homogeneity of an image. Unlike Homogeneity1, IDN normalizes the difference between the neighboring intensity values by dividing over the total number of discrete intensity values. |
| 19 | Inverse Variance | Inverse-variance weighting is a method of aggregating two or more random variables to minimize the variance of the weighted average. |
| 20 | Maximum Probability | Maximum Probability is occurrences of the most predominant pair of neighboring intensity values. |
| 21 | Sum Entropy | The sum of neighborhood intensity value differences. |
| 22 | Sum Squares | A measure in the distribution of neigboring intensity level pairs about the mean intensity level in the GLCM. |

***Group 4. Gray-Level Size Zone Matrix (GLSZM) features***

A Gray Level Size Zone (GLSZM) quantifies gray level zones in an image. A gray level zone is defined as the number of connected voxels that share the same gray level intensity. A voxel is considered connected if the distance is 1 according to the infinity norm (26-connected region in a 3D, 8-connected region in 2D). In a gray level size zone matrix $P(i,j)$ the ${(i,j)}^{th}$ element equals the number of zones with gray level ii and size jj appear in image. Contrary to GLCM and GLRLM, the GLSZM is rotation independent, with only one matrix calculated for all directions in the VOI.

**Table S5.** Descriptions of all features for GLSZM characteristics.

| No. | Feature names | Descriptions |
| --- | --- | --- |
| 1 | Gray Level Non-Uniformity (GLN) | GLN measures the variability of gray-level intensity values in the image, with a lower value indicating more homogeneity in intensity values. |
| 2 | Gray Level Non-Uniformity Normalized (GLNN) | GLNN measures the variability of gray-level intensity values in the image, with a lower value indicating a greater similarity in intensity values. This is the normalized version of the GLN formula. |
| 3 | Gray Level Variance (GLV) | GLV measures the variance in gray level intensities for the zones. |
| 4 | High Gray Level Zone Emphasis (HGLZE) | HGLZE measures the distribution of the higher gray-level values, with a higher value indicating a greater proportion of higher gray-level values and size zones in the image. |
| 5 | Large Area Emphasis (LAE) | LAE is a measure of the distribution of large area size zones, with a greater value indicative of larger size zones and more coarse textures. |
| 6 | Large Area High Gray Level Emphasis (LAHGLE) | LAHGLE measures the proportion in the image of the joint distribution of larger size zones with higher gray-level values. |
| 7 | Large Area Low Gray Level Emphasis (LALGLE) | LALGLE measures the proportion in the image of the joint distribution of larger size zones with lower gray-level values. |
| 8 | Low Gray Level Zone Emphasis (LGLZE) | LGLZE measures the distribution of lower gray-level size zones, with a higher value indicating a greater proportion of lower gray-level values and size zones in the image. |
| 9 | Size Zone Non-Uniformity (SZN) | SZN measures the variability of size zone volumes in the image, with a lower value indicating more homogeneity in size zone volumes. |
| 10 | Size Zone Non-Uniformity Normalized (SZNN) | SZNN measures the variability of size zone volumes throughout the image, with a lower value indicating more homogeneity among zone size volumes in the image. This is the normalized version of the SZN formula. |
| 11 | Small Area Emphasis (SAE) | SAE is a measure of the distribution of small size zones, with a greater value indicative of smaller size zones and more fine textures. |
| 12 | Small Area High Gray Level Emphasis (SAHGLE) | SAHGLE measures the proportion in the image of the joint distribution of smaller size zones with higher gray-level values. |
| 13 | Small Area Low Gray Level Emphasis (SALGLE) | SALGLE measures the proportion in the image of the joint distribution of smaller size zones with lower gray-level values. |
| 14 | Zone Entropy | Measures the uncertainty/randomness in the distribution of zone sizes and gray levels. A higher value indicates more heterogeneity in the texture patterns. |
| 15 | Zone Percentage | Measures the coarseness of the texture by taking the ratio of number of zones and number of voxels in the VOI. |
| 16 | Zone Variance | Measures the variance in zone size volumes for the zones. |

***Group 5. Gray-Level Run-Length Matrix (GLRLM) features***

Run length metrics quantify gray level runs in an image. A gray level run is defined as the length in number of pixels, of consecutive pixels that have the same gray level value. In a gray level run length matrix $p(i,j|\theta)$, the $(i,j)$th element describes the number of times $j$ a gray level $i$ appears consecutively in the direction specified by $\theta$, and $N_{g}$ is the number of discrete gray level intensities. In this study, a GLRL matrix was computed for every of the 13 directions in three dimensions, from which the below textural features were derived. Each 3D GLRL feature was then calculated as the mean of the feature values for each of the 13 directions.

**Table S6.** Descriptions of all features for GLRLM characteristics.

| No. | Feature names | Descriptions |
| --- | --- | --- |
| 1 | Gray Level Non Uniformity (GLN) | GLN measures the similarity of gray-level intensity values in the image, where a lower GLN value correlates with a greater similarity in intensity values. |
| 2 | Gray Level NonUniformity Normalized (GLNN) | GLNN measures the similarity of gray-level intensity values in the image, where a lower GLNN value correlates with a greater similarity in intensity values. This is the normalized version of the GLN formula. |
| 3 | Gray Level Variance (GLV) | GLV measures the variance in gray level intensity for the runs. |
| 4 | High Gray Level Run Emphasis (LGLRE) | HGLRE measures the distribution of the higher gray-level values, with a higher value indicating a greater concentration of high gray-level values in the image. |
| 5 | Long Run Emphasis | LRE is a measure of the distribution of long run lengths, with a greater value indicative of longer run lengths and more coarse structural textures. |
| 6 | Long Run High Gray Level Emphasis (LRHGLRE) | LRHGLRE measures the joint distribution of long run lengths with higher gray-level values. |
| 7 | Long Run Low Gray Level Emphasis (LRLGLRE) | LRLGLRE measures the joint distribution of long run lengths with lower gray-level values. |
| 8 | Low Gray Level Run Emphasis (LGLRE) | LGLRE measures the distribution of low gray-level values, with a higher value indicating a greater concentration of low gray-level values in the image. |
| 9 | Run Entropy | Measures the uncertainty/randomness in the distribution of run lengths and gray levels. A higher value indicates more heterogeneity in the texture patterns. |
| 10 | Run Length Non-Uniformity (RLN) | RLN measures the similarity of run lengths throughout the image, with a lower value indicating more homogeneity among run lengths in the image. |
| 11 | Run Length Non-Uniformity Normalized | RLNN measures the similarity of run lengths throughout the image, with a lower value indicating more homogeneity among run lengths in the image. This is the normalized version of the RLN formula. |
| 12 | Run Percentage | Measures the coarseness of the texture by taking the ratio of number of runs and number of voxels in the VOI. |
| 13 | Run Variance | A measure of the variance in runs for the run lengths. |
| 14 | Short Run Emphasis (SRE) | SRE is a measure of the distribution of short run lengths, with a greater value indicative of shorter run lengths and more fine textural textures. |
| 15 | Short Run High Gray Level Emphasis (SRHGLE) | SRHGLE measures the joint distribution of shorter run lengths with higher gray-level values. |
| 16 | Short Run Low Gray Level Emphasis (SRLGLE) | SRLGLE measures the joint distribution of shorter run lengths with lower gray-level values. |

***Group 6. Gray-Level Dependence Matrix (GLDM) features***

A Gray Level Dependence Matrix (GLDM) quantifies gray level dependencies in an image. A gray level dependency is defined as a the number of connected voxels within distance δδ that are dependent on the center voxel. A neighbouring voxel with gray level jj is considered dependent on center voxel with gray level $i$ if $\left| i-j \right|\leq\alpha$. In a gray level dependence matrix $P(i,j)$ the ${(i,j)}^{th}$element describes the number of times a voxel with gray level $i$ with $j$ dependent voxels in its neighbourhood appears in image.

**Table S7.** Descriptions of all features for GLDM characteristics.

| No. | Feature names | Descriptions |
| --- | --- | --- |
| 1 | Dependence Entropy | A measure of the randomness/variability in dependence size in the image. |
| 2 | Dependence Non-Uniformity (DN) | Measures the similarity of dependence throughout the image, with a lower value indicating more homogeneity among dependencies in the image. |
| 3 | Dependence Non-Uniformity Normalized (DNN) | Measures the similarity of dependence throughout the image, with a lower value indicating more homogeneity among dependencies in the image. This is the normalized version of the DLN formula. |
| 4 | Dependence Variance | Measures the variance in dependence size in the image. |
| 5 | Gray Level Non-Uniformity (GLN) | Measures the similarity of gray-level intensity values in the image, where a lower GLN value correlates with a greater similarity in intensity values. |
| 6 | Gray Level Variance (GLV) | Measures the variance in grey level in the image. |
| 7 | High Gray Level Emphasis (HGLE) | Measures the distribution of the higher gray-level values, with a higher value indicating a greater concentration of high gray-level values in the image. |
| 8 | Large Dependence Emphasis (LDE) | A measure of the distribution of large dependencies, with a greater value indicative of larger dependence and more homogeneous textures. |
| 9 | Large Dependence High Gray Level Emphasis (LDHGLE) | Measures the joint distribution of large dependence with higher gray-level values. |
| 10 | Large Dependence Low Gray Level Emphasis (LDLGLE) | Measures the joint distribution of large dependence with lower gray-level values. |
| 11 | Low Gray Level Emphasis (LGLE) | Measures the distribution of low gray-level values, with a higher value indicating a greater concentration of low gray-level values in the image. |
| 12 | Small Dependence Emphasis (SDE) | A measure of the distribution of small dependencies, with a greater value indicative of smaller dependence and less homogeneous textures. |
| 13 | Small Dependence High Gray Level Emphasis (SDHGLE) | Measures the joint distribution of small dependence with higher gray-level values. |
| 14 | Small Dependence Low Gray Level Emphasis (SDLGLE) | Measures the joint distribution of small dependence with lower gray-level values. |

1. **The important of the selected features of models**

For the clinical model, the feature importance was shown in Fig.S1. The top 3 important clinical factors were the occurrence of fatigue, age and the occurrence of cough. The feature importance of the quantifying model was shown in Fig. S2. The frequency of the pleural thickening, consolidation lesion and ground glass lesion were the top 3 importance features. For the radiological model, as were shown in Fig. S3, the frequency occurrence of paving stone, position at periphery, and subpleural line were importance for the discrimination of the COVID-19 from other pneumonia. Finally, the feature importance of the radiomic model were shown in Fig. S4. The most important feature was Zone Entropy of glszm on the wavelet filtered image, indicating the heterogeneneity in the texture patterns. The shape of the lesion was also important, and the Minor Axis Length and Maximum 2D Diameter Slice were the second and third most important radiomic features.

1. **The radiomics guideline**

To ensure the completeness of the description of the study workflow, the radiomics guideline[[1](#_ENREF_1), [2](#_ENREF_2)] were used as references. The corresponding page in our manuscript were also listed.

Table S8. The radiomics guideline

| Category | Guideline. | Page |
| --- | --- | --- |
| General |  |  |
| Image acquisition | Acquisition protocols and scanner parameters such as equipment vendor, reconstruction algorithms and filters, field of view and acquisition matrix dimensions, MRI sequence parameters, PET acquisition time and injected dose, CT x-ray energy (kVp), and exposure (mAs). | 6 |
| Volumetric analysis | Specification of whether imaging volumes were analyzed as separate images (2-dimensional) or as fully-connected volumes (3-dimensional). | 3 |
| Workflow structure | Sequence of processing steps leading to extraction of features. | 6 |
| Software | Software type and version of code used for computation of features. | 9 |
| Image preprocessing |  |  |
| Conversion | How data were converted from input images (e.g., conversion of PET activity counts to SUV and calculation of ADC maps from raw diffusion-weighted MRI signal). | NA |
| Processing | Image-processing steps after acquisition (e.g., noise filtering, intensity nonuniformity correction in MRI, and partial-volume effect corrections). | 7 |
| ROI segmentation[*****](https://jnm.snmjournals.org/content/59/2/189#fn-2)[**†**](https://jnm.snmjournals.org/content/59/2/189#fn-3) | How ROIs were delineated in images (e.g., software or algorithms used, number of persons and their level of expertise [specialty, experience], method of reaching consensus, and mode [automatic or semiautomatic]). | 6 |
| Interpolation |  |  |
| Voxel dimensions | Original and interpolated voxel dimensions. | 7 |
| Image interpolation method | Method used for interpolating voxel values (e.g., linear, cubic, or spline) and for aligning original and interpolated grids. | 7 |
| Intensity rounding | Rounding procedures for noninteger interpolated gray levels (if applicable) (e.g., rounding of Hounsfield units in CT images after interpolation). | NA |
| ROI interpolation method | Methods used for interpolating ROI masks and for aligning original and interpolated grids. | NA |
| ROI partial volume | Minimum partial-volume fraction required to include an interpolated mask voxel in the interpolated ROI (if applicable) (e.g., minimum partial-volume fraction of 0.5 when using linear interpolation). | NA |
| ROI resegmentation |  |  |
| Inclusion/exclusion criteria | Criteria for inclusion or exclusion of voxels from the ROI intensity mask (if applicable) (e.g., exclusion of voxels with Hounsfield unit values outside predefined range inside the ROI intensity mask on CT images). | NA |
| Image discretization |  |  |
| Discretization method | Method used for discretizing image intensities before feature extraction (e.g., fixed bin number, fixed bin width, and histogram equalization). | 7 |
| Discretization parameters | Parameters for image discretization (e.g., number of bins, bin width, and minimal value of discretization range). | 7 |
| Feature calculation |  |  |
| Feature set | Description and formulas of all calculated features. | 7 |
| Feature parameters | Settings for calculation of features (e.g., voxel connectivity, with or without merging by slice, and with or without merging directional texture matrices). | 7 and supplementary material |
| Calibration |  |  |
| Image-processing steps | Specification of which image-processing steps match benchmarks of the IBSI. | 7 |
| Feature calculation | Specification of which feature calculations match benchmarks of the IBSI. | 7 and supplementary material |

1. Vallières M, Zwanenburg A, Badic B, Cheze Le Rest C, Visvikis D, Hatt M: Responsible Radiomics Research for Faster Clinical Translation. J Nucl Med 2018, 59**:**189-193.

2. Lambin P, Leijenaar RTH, Deist TM, Peerlings J, de Jong EEC, van Timmeren J, Sanduleanu S, Larue R, Even AJG, Jochems A, et al: Radiomics: the bridge between medical imaging and personalized medicine. Nat Rev Clin Oncol 2017, 14**:**749-762.
