# Supplementary figures and images for: Machine learning-based CT radiomics model distinguishes COVID-19 from non-COVID-19 pneumonia
Source: BMC Infect Dis. 2021 Sep 8;21:931. doi: 10.1186/s12879-021-06614-6 (PMC8424152; doi:10.1186/s12879-021-06614-6)

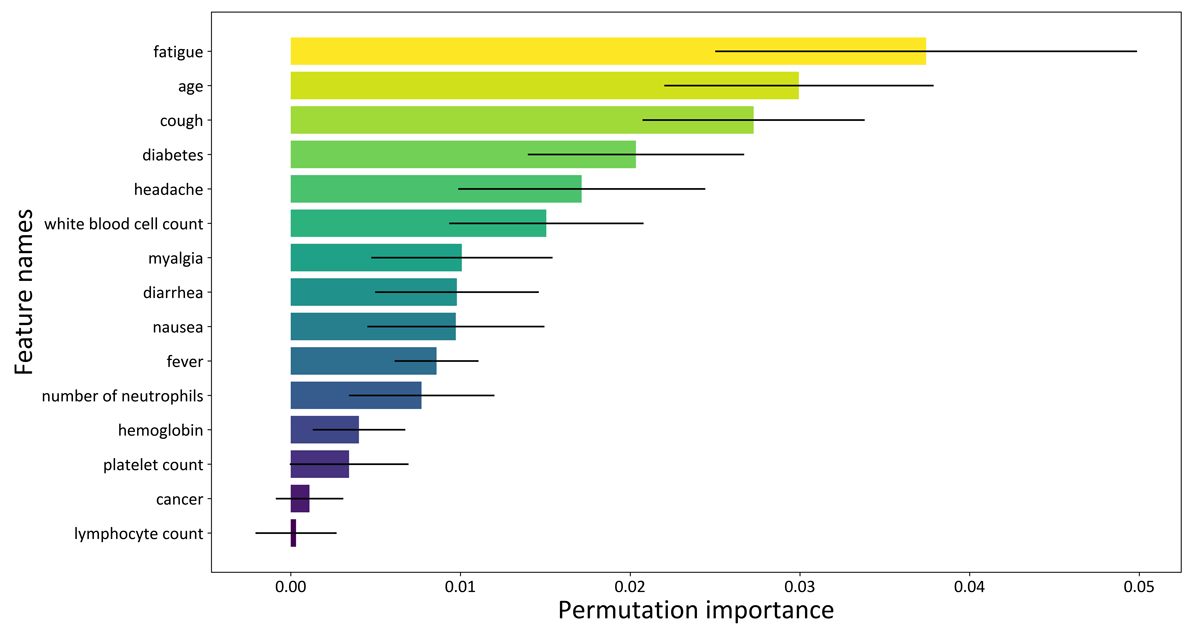

Supplement: Supplementary file 2 — Additional file 2. For the clinical model, the feature importance was shown. The top 3 important clinical factors were the occurrence of fatigue, age and the occurrence of cough. [file 12879_2021_6614_MOESM2_ESM.tif]

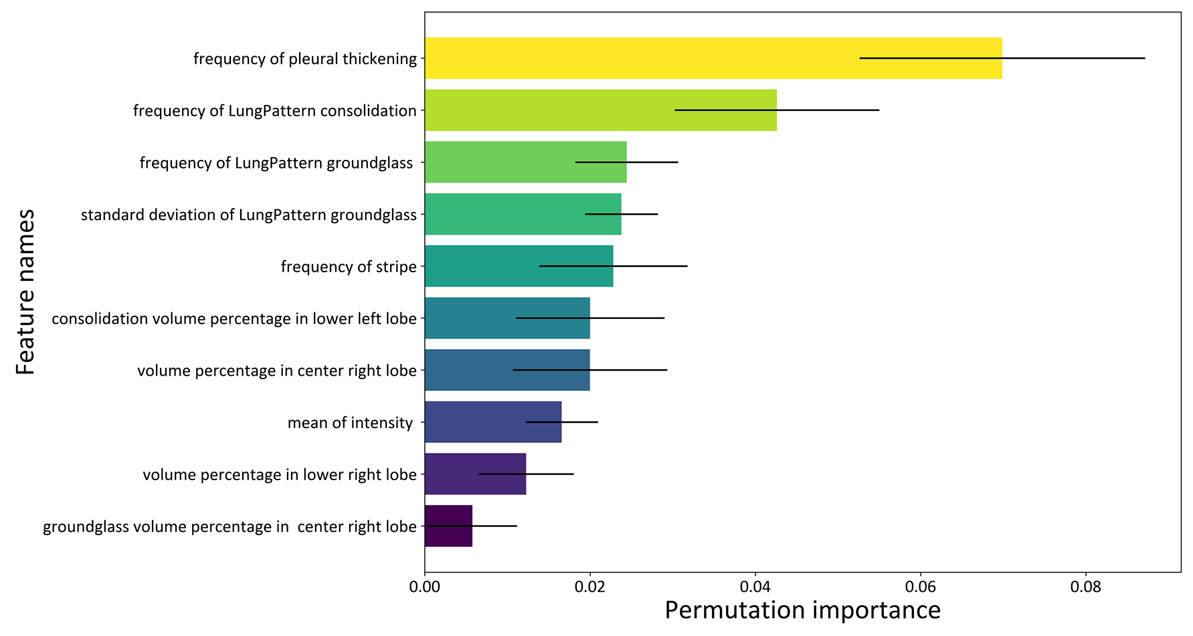

Supplement: Supplementary file 3 — Additional file 3. The feature importance of the quantifying model was shown. The frequency of the pleural thickening, consolidation lesion and ground glass lesion were the top 3 importance features. [file 12879_2021_6614_MOESM3_ESM.tif]

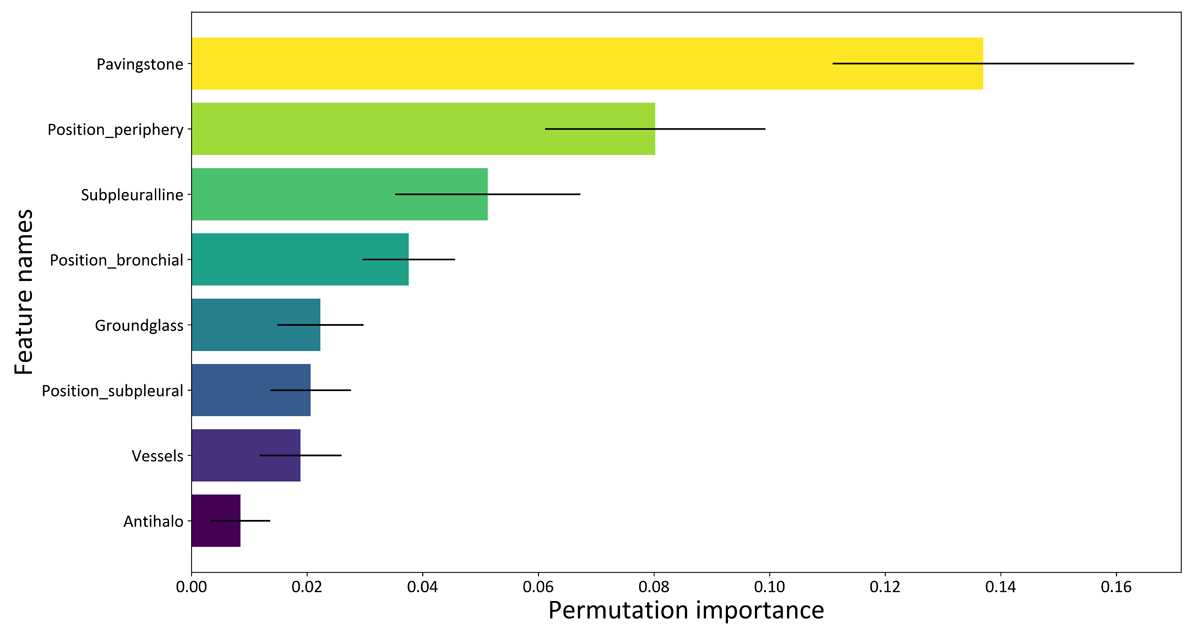

Supplement: Supplementary file 4 — Additional file 4. The radiological model was shown. The frequency occurrence of paving stone, position at periphery, and subpleural line were importance for the discrimination of the COVID-19 from other pneumonia. [file 12879_2021_6614_MOESM4_ESM.tif]

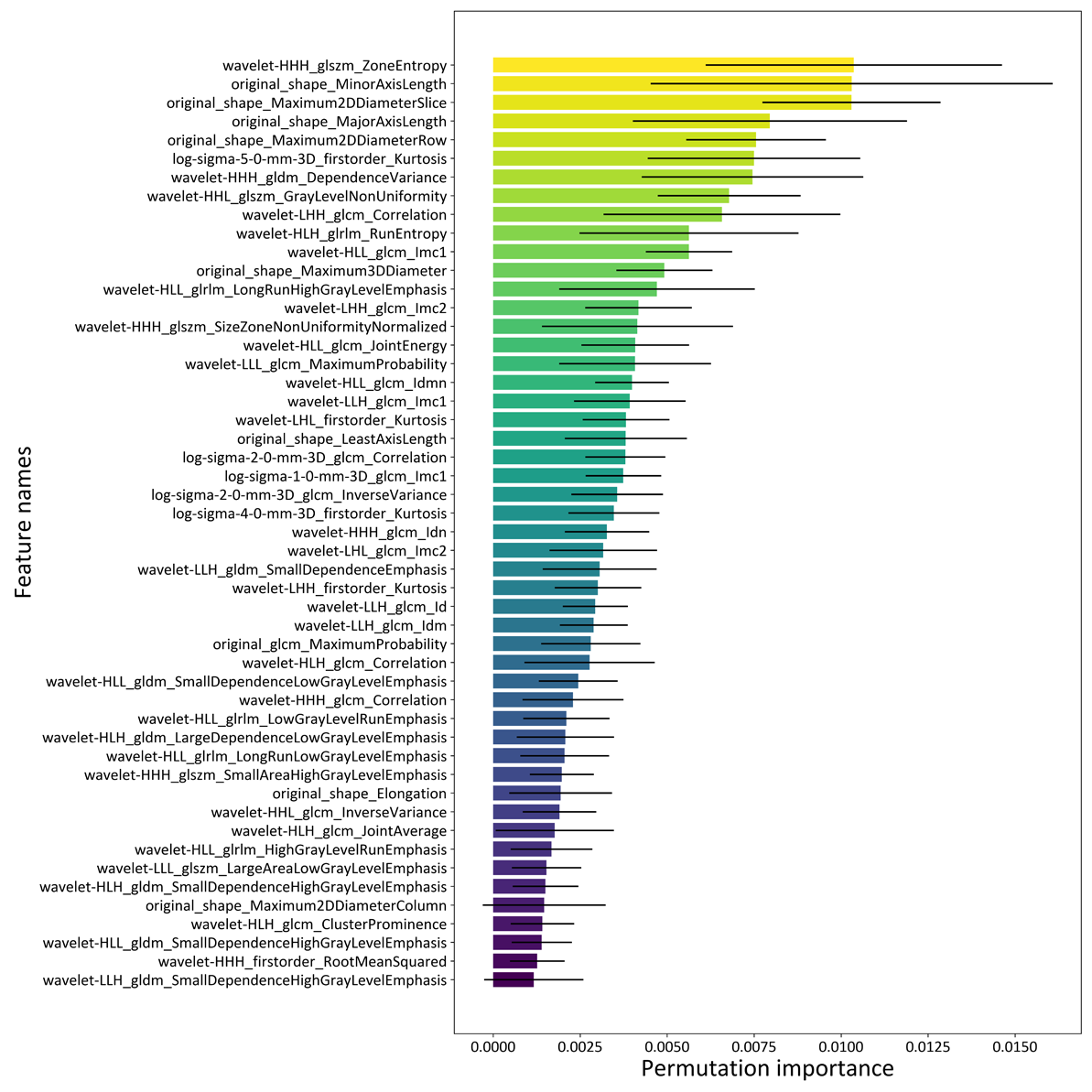

Supplement: Supplementary file 5 — Additional file 5. The feature importance of the radiomic model was shown. The most important feature was Zone Entropy of glszm on the wavelet filtered image, indicating the heterogeneneity in the texture patterns. The shape of the lesion was also important, and the Minor Axis Length and Maximum 2D Diameter Slice were the second and third most important radiomic features. [file 12879_2021_6614_MOESM5_ESM.tif]
